# Interleukins in Major Depressive Disorder: Lessons From Autoimmune Diseases and Pathways to Clinical Translation

**DOI:** 10.1002/cns.70791

**Published:** 2026-02-17

**Authors:** Adnan Akif, Mohammad Fahim Kadir, Md. Rabiul Islam

**Affiliations:** ^1^ Department of Physiology & Cell Biology, School of Medicine University of Nevada Reno Nevada USA; ^2^ Department of Pharmacology Campbell University School of Osteopathic Medicine Buies Creek North Carolina USA; ^3^ School of Pharmacy BRAC University Dhaka Bangladesh

**Keywords:** autoimmune diseases, depression, inflammation, interleukin, major depressive disorder, rheumatoid arthritis

## Abstract

**Background:**

Major Depressive Disorder (MDD) is a leading cause of disability, and limitations of the monoamine hypothesis have driven the exploration of complementary models, including the inflammatory hypothesis. This hypothesis suggests that in a subset of patients, immune dysregulation, especially involving interleukins (ILs), contributes to depression. This review compares IL dysregulation in MDD and autoimmune diseases to identify common and unique inflammatory mechanisms for treatment.

**Results and Conclusion:**

Some MDD patients exhibit chronic, low‐grade inflammation both systemically (in blood) and centrally (in CSF and brain). They show elevated pro‐inflammatory IL‐6, IL‐1β, and IL‐18, with insufficient anti‐inflammatory IL‐10. This immune dysregulation affects key neurobiological processes (monoamine metabolism, HPA axis, neurogenesis), linking inflammation to depressive symptoms. MDD and autoimmune diseases share inflammatory mediators and signaling pathways, supporting these as therapeutic targets; however, MDD's inflammation is low‐grade and innate driven, whereas autoimmune diseases have high‐grade, adaptive immune responses to specific antigens. Successful anti‐IL therapies in autoimmune conditions provide a roadmap for treating inflammation‐driven depression. Translating these findings to practice requires a precision immune‐psychiatry approach. Biomarkers like C‐reactive protein and IL‐6 can identify an inflammatory depression subtype, and patients may benefit from targeted immunomodulatory strategies, repurposed biologics, novel small molecules, or lifestyle interventions particularly if standard antidepressants fail.

## Graphical abstract

Major Depressive Disorder (MDD) is a leading cause of disability, and limitations of the monoamine hypothesis have spurred interest in an inflammatory model of depression. Immune dysregulation, especially involving interleukins (ILs), contributes to depression. This affects key neurobiological processes, linking inflammation to depressive symptoms. Depressive disorder and autoimmune diseases share inflammatory mediators and signaling pathways, supporting anti‐IL therapies as therapeutic targets.

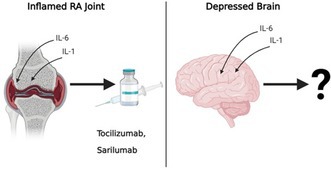

## 1 Introduction

Major Depressive Disorder (MDD) is prevalent and disabling; the World Health Organization estimates that ~5% of adults worldwide (~280 million people) are affected [1]. Although the monoamine hypothesis guided effective antidepressants, it does not fully explain MDD's heterogeneity or variable treatment response [2]. Experimental serotonin depletion (e.g., tryptophan depletion) does not reliably induce depression in healthy individuals nor consistently worsen symptoms in unmedicated patients, challenging a simple “low serotonin causes MDD” model [3]. These gaps have driven complementary frameworks, including immune and inflammatory models of depression.

Building on limitations of the monoamine model, the inflammatory/cytokine hypothesis proposes that immune dysregulation contributes to MDD in a biologically defined subgroup. Early work demonstrated increased production of cytokines such as IL‐1β and IL‐6 in MDD [4, 5], and interleukins can influence neurotransmitter metabolism, HPA‐axis function, and neuroplasticity [6]. Key gaps remain why inflammation marks only a subset of patients, how specific interleukins map onto symptom domains, and why anti‐inflammatory trials are mixed—highlighting the need for biomarkers and stratified designs. Immune–brain interactions are bidirectional, as depressive states and psychosocial stress can also amplify inflammation via neuroendocrine and autonomic pathways [7, 8].

This review posits that many of these knowledge gaps can be addressed by adopting a comparative approach. Autoimmune diseases like RA offer clearer examples of interleukin‐driven pathology and provide a “proof‐of‐concept” for successful, highly specific immunomodulatory therapies [9, 10]. The primary aim of this review is to provide an exhaustive analysis of the role of interleukins in the pathophysiology of MDD. By comparing and contrasting the interleukin dysregulation observed in MDD with that of hallmark autoimmune diseases—RA—this review will: (1) synthesize the evidence for the involvement of specific interleukins in MDD; (2) elucidate the shared and distinct immunological features between MDD and these comparator diseases; (3) critically evaluate the lessons learned from successful and unsuccessful immunomodulatory therapies; and (4) discuss the profound diagnostic and therapeutic implications for MDD, charting a course toward the clinical translation of these findings and the dawn of a new era of precision immunopsychiatry. This comparative framework allows for the identification of shared pathways, the delineation of MDD‐specific inflammatory signatures, and the generation of a clinical and therapeutic roadmap to guide the translation of immunotherapies into psychiatry.

## 2 Interleukin Dysregulation in MDD

The “inflammatory subtype” of MDD is not merely a theoretical construct but is supported by a growing and robust body of clinical evidence [11]. This evidence spans from measurements of circulating inflammatory molecules in the blood to direct visualization of neuroinflammatory processes in the living human brain.

### 2.1 Evidence for Systemic Inflammation in MDD: Peripheral Cytokine Analyses

The most extensive evidence for immune dysregulation in MDD comes from the analysis of peripheral blood. Numerous studies and multiple meta‐analyses have consistently demonstrated that individuals with MDD exhibit a profile of chronic, low‐grade systemic inflammation. The most reliably elevated pro‐inflammatory biomarkers are interleukin‐6 (IL‐6), tumor necrosis factor‐alpha (TNF−α), and the downstream hepatic acute‐phase reactant C‐reactive protein (CRP) [11]. In addition to this core triad, elevations in other pro‐inflammatory cytokines, including IL−1β, interleukin‐12 (IL‐12), and interleukin‐18 (IL‐18), are also frequently reported [12]. This consistent pattern of elevated peripheral markers provides strong support for the hypothesis that at least a significant subgroup of MDD patients exists in a state of persistent systemic inflammation. “Low‐grade inflammation” may refer to modest, chronic elevations in inflammatory markers (e.g., hs‐CRP and circulating cytokines) in the absence of acute infection [13, 14]. However, the term “low‐grade” inflammation requires a more precise definition.

### 2.2 Evidence for Neuroinflammation in MDD: CNS‐Specific Data

Crucially, this inflammatory signature is not confined to the periphery. Multiple lines of evidence indicate that the inflammatory process extends into, or is mirrored within, the central nervous system itself.

#### 2.2.1 Cerebrospinal Fluid (CSF) Studies

CSF provides a more direct window into the biochemical environment of the brain. Studies of cytokine levels in the CSF of MDD patients have revealed significant elevations of pro‐inflammatory mediators, most notably IL‐6 and the chemokine interleukin‐8 (IL‐8), compared to healthy controls [15]. These findings confirm that the inflammatory state is present within the neuroaxis and is not merely a peripheral phenomenon.

#### 2.2.2 Post‐Mortem Brain Studies

Investigations of post‐mortem brain tissue from individuals who had MDD or died by suicide have provided histological evidence of neuroinflammation. These studies have documented increased expression of pro‐inflammatory cytokines like TNF −α and markers of glial activation, particularly activated microglia, in key mood‐regulating brain regions such as the prefrontal cortex and anterior cingulate cortex [16, 17]. While not all studies agree, with some showing no changes or even decreases in certain glial markers, the balance of evidence points toward a neuroinflammatory process in these critical areas [18].

#### 2.2.3 Positron Emission Tomography (PET) Imaging

Multiple PET‐TSPO (translocator protein) studies and comprehensive meta‐analysis have consistently shown significantly increased translocator protein (TSPO) binding (a protein that is upregulated on the mitochondrial membrane of activated microglia and astrocytes) in individuals with MDD [19, 20]. This increase is not diffuse but is localized to brain regions central to mood regulation, including the anterior cingulate cortex, prefrontal cortex, insula, and hippocampus, providing direct visual proof of neuroinflammation in the brains of depressed patients [21].

#### 2.2.4 Neural‐Circuit Relevance and Symptom Domains

Converging neuroimaging and mechanistic data suggest that inflammation maps onto partially distinct symptom clusters via effects on specific circuits. Salience and affective‐pain networks (anterior cingulate cortex and insula) and threat circuitry (amygdala) are linked to depressed mood and anxiety, whereas hippocampal involvement aligns with cognitive dysfunction [22, 23, 24]. Inflammatory signaling also targets corticostriatal reward pathways; experimental immune activation reduces ventral striatum reward responsiveness, consistent with anhedonia, and inflammatory markers are associated with altered basal ganglia glutamate/dopamine function, aligning with fatigue and psychomotor slowing [25, 26, 27]. At the molecular level, cytokines such as IL‐1β and IL‐6 can engage indoleamine‐2,3‐dioxygenase and shift tryptophan metabolism toward kynurenine metabolites (e.g., quinolinic acid), promoting glutamatergic hyperactivity and impaired neuroplasticity [28, 29]. The key roles of interleukins in the pathophysiology of MDD are summarized in Table 1.

**TABLE 1:** Summary of key interleukins in major depressive disorder.

Interleukins	Role	Direction in MDD	Key findings	Mechanistic/Pathway notes	Clinical/Imaging correlates	References
IL‐1β	Pro‐inflammatory	↑ (peripheral)	Elevated levels associated with MDD; activates HPA axis; inhibits adult hippocampal neurogenesis; early‐life adversity may prime IL‐1β‐driven subtype.	HPA axis activation; reduced neurogenesis; stress‐primed immune response.	Etiological subgroups are linked to childhood trauma.	[30, 31, 32]
IL‐6	Pro‐inflammatory	↑ (peripheral & CSF)	Most consistently identified biomarker; elevations relate to greater symptom severity and antidepressant resistance; dysregulated diurnal rhythm; linked to cortical morphology.	Disrupted diurnal secretion; neuroinflammatory effects.	Reduced prefrontal cortex thickness; worse severity; treatment resistance.	[33, 34, 35]
IL‐18	Pro‐inflammatory (IL‐1 family)	↑ (peripheral)	Inflammasome‐activated; elevated in MDD; links peripheral inflammation to brain function.	Inflammasome activation under cellular stress.	Higher serum associated with decreased posterior cingulate activity (DMN).	[36, 37]
IL‐10	Anti‐inflammatory	Mixed (↓ in some & ↑ in others)	Evidence for immunoregulatory failure; elevated IL‐6/IL‐10 ratio; loss of normal positive IL‐6↔IL‐10 coupling seen in controls.	Counter‐regulatory to IL‐6; failure of negative feedback loop.	Dysregulated IL‐6/IL‐10 balance relates to inflammatory burden and possibly symptom persistence.	[38, 39, 40]
IL‐4	Anti‐inflammatory (Th2)	Mixed (often ↓)	Th2 cytokine; evidence mixed overall; some data indicate decreased IL‐4 relates to suicidality.	Counterbalances Th1/Th17 responses.	Lower levels are linked to suicidal behavior in some cohorts.	[41]

*Note:* Arrows (↑, ↓) indicate increased or decreased levels in MDD.

Abbreviations: CSF, Cerebrospinal Fluid; DMN, Default Mode Network; HPA, Hypothalamic–Pituitary–Adrenal; IL, Interleukin; MDD, Major Depressive Disorder; Th2, T‐helper type 2.

### 2.3 Sources of IL Dysregulation in MDD

The chronic, low‐grade inflammation observed in MDD is typically not the result of an acute infection. Instead, it appears to be a multifactorial process driven by a convergence of environmental, behavioral, and biological factors that place a cumulative inflammatory load on the body.

#### 2.3.1 Psychosocial Stress

Chronic psychological stress is a primary driver, activating the sympathetic nervous system and the HPA axis, both of which can stimulate immune cells to produce pro‐inflammatory cytokines [7, 42].

#### 2.3.2 Gut Microbiome Dysbiosis

An unhealthy balance of gut bacteria can lead to increased intestinal permeability, or “leaky gut.” This allows bacterial products, such as lipopolysaccharide (LPS), to translocate from the gut into the systemic circulation [43]. LPS is a powerful trigger for the innate immune system, providing a persistent, low‐level inflammatory stimulus that contributes to the systemic inflammation seen in MDD [44].

#### 2.3.3 Genetic Vulnerabilities

An individual's genetic makeup can influence their inflammatory response. Polymorphisms in the genes that code for cytokines (like IL‐6) or their receptors can predispose a person to mount a more robust or prolonged inflammatory reaction to a given stressor [45].

#### 2.3.4 Early Life Adversity

Exposure to trauma or severe stress in childhood can have life‐long consequences, including the “programming” of the immune system toward a chronic pro‐inflammatory state [30, 46].

#### 2.3.5 Lifestyle and Comorbidities

**FIGURE 1:**
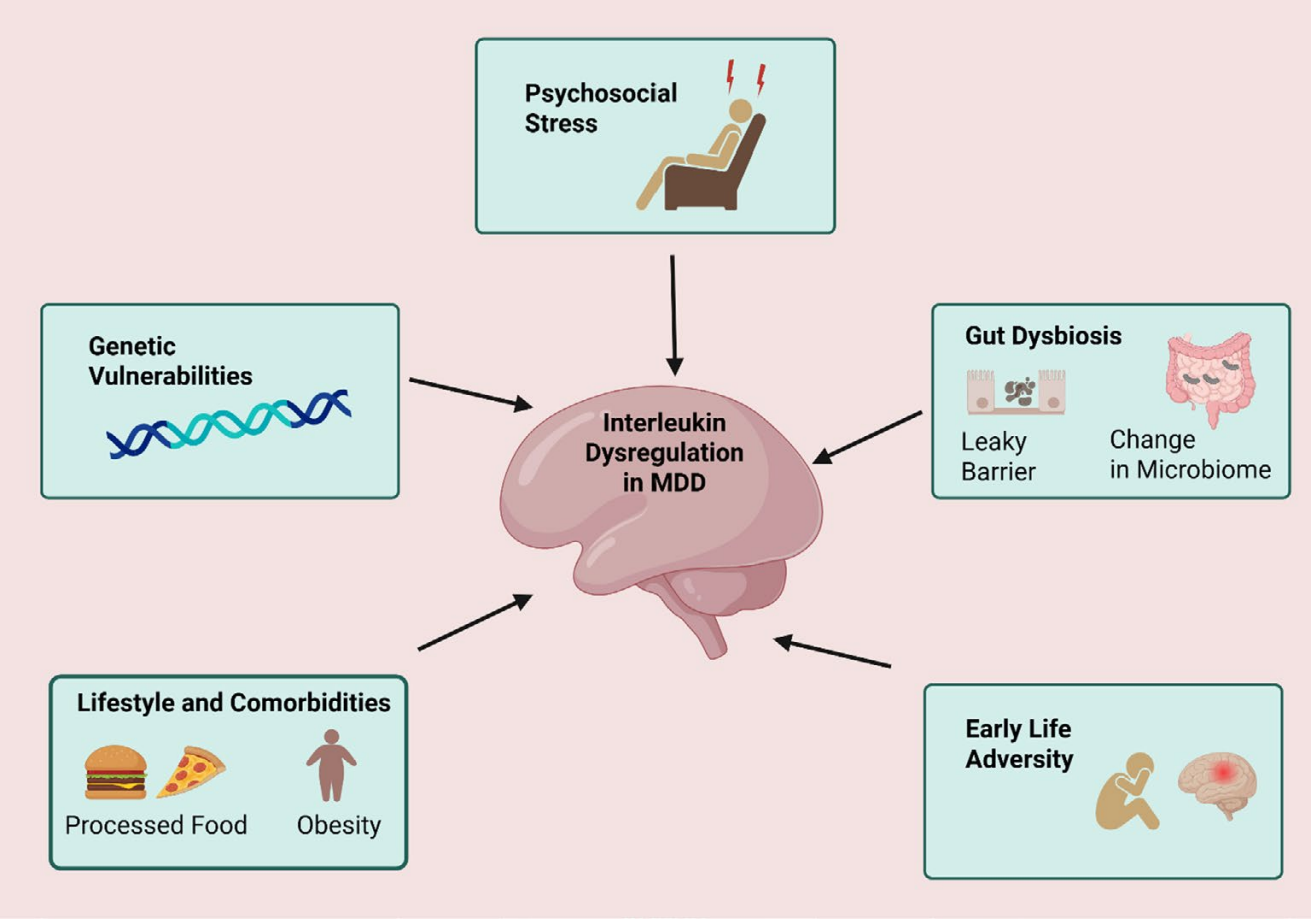
Proposed contributors to dysregulated interleukin (IL) signaling in MDD. The diagram illustrates five primary sources that can lead to abnormal pro‐ and anti‐inflammatory IL levels, fostering a neuroinflammatory state implicated in the development and persistence of MDD.

Modern lifestyle factors and associated medical conditions are major contributors. Obesity, metabolic syndrome, and type 2 diabetes are themselves states of chronic inflammation and are highly comorbid with MDD [47]. It is likely that these conditions and depression share and mutually reinforce a common inflammatory milieu. Figure 1 demonstrates the proposed contributors to dysregulated IL signaling in MDD.

## 3 Comparative Analysis: Interleukins in MDD Versus Autoimmune Disorders

### 3.1 Rheumatoid Arthritis (RA)

#### 3.1.1 RA Pathogenesis and IL Profile

RA is a systemic autoimmune disease characterized by chronic inflammation of the synovial joints, leading to progressive cartilage and bone destruction [48]. The pathogenesis involves a robust adaptive immune response against self‐antigens within the joint, driven by autoreactive T‐cells and B‐cells [49]. The cytokine milieu in the RA synovium is intensely pro‐inflammatory. TNF−α and IL‐6 are considered the two central pillars of RA pathogenesis, driving inflammation, synovial cell proliferation, and joint erosion [50]. Interleukin‐1 beta (IL−1β) is another key mediator of inflammation and damage [51]. Furthermore, the Th17 pathway, promoted by IL‐23 and characterized by the production of IL‐17, is critically involved in perpetuating the inflammatory cycle and promoting osteoclast activity [52, 53].

#### 3.1.2 Contrasts and Overlaps With MDD

The most striking contrast is the magnitude of inflammation; RA is a high‐grade inflammatory disease, whereas MDD is associated with low‐grade inflammation. RA is also clearly driven by an adaptive immune response against specific joint‐related autoantigens, a feature absents in MDD, where innate immunity appears more dominant. However, the overlaps are highly informative. Both conditions share IL‐6, IL−1β, and TNF−α as key mediators and utilize common downstream signaling pathways like NF‐κB and JAK–STAT [54, 55]. Clinically, the overlap is profound: depression is highly comorbid with RA and patients suffer from severe fatigue and anhedonia, symptoms that are core to MDD [56].

#### 3.1.3 Therapeutic Implications From RA

The history of RA treatment provides a powerful playbook for immunopsychiatry. The development of biologic drugs that specifically target key cytokines—most notably anti‐TNF agents (e.g., infliximab, adalimumab) and anti‐IL‐6 receptor agents (e.g., tocilizumab, sarilumab)—has revolutionized the management of RA, moving it from a progressively disabling disease to a manageable chronic condition [57]. These successes not only validate these specific cytokines and pathways as druggable targets but also demonstrate the feasibility and benefit of a targeted immunomodulatory approach, providing a clear roadmap for trials in depression.

### 3.2 Multiple Sclerosis (MS)

#### 3.2.1 MS Pathogenesis and IL Profile

MS is a chronic autoimmune disease of the CNS defined by inflammation, demyelination, and neurodegeneration [58]. The pathology is driven by the infiltration of autoreactive T‐cells and B‐cells across the BBB, which then mount an attack on the myelin sheath of neurons [59]. MS is classically considered a Th1‐ and Th17‐mediated disease. The characteristic interleukin profile includes high levels of interferon‐gamma (IFN−γ) from Th1 cells and IL‐17 from Th17 cells. The cytokines that drive these responses, IL‐12 and IL‐23, are also central to the pathology, along with IL−1β and IL‐6 [60]. In contrast, the anti‐inflammatory cytokine IL‐10 appears to be protective, with its function often impaired in MS patients [61].

#### 3.2.2 Contrasts and Overlaps With MDD

Unlike the systemic low inflammation of MDD, MS involves high‐grade inflammation specifically compartmentalized within the CNS. The IL profile is also more specifically skewed toward Th1/Th17 pathways targeting myelin antigens than the more mixed profile seen in MDD [62]. However, the overlaps are again significant. IL−1β, IL‐6, and IL‐17 are implicated in both conditions [63, 64]. The clinical overlap is particularly stark: depression is extremely common in MS, and debilitating fatigue is a hallmark symptom [65]. This provides a direct clinical link between profound CNS inflammation and the development of mood disorders.

#### 3.2.3 Therapeutic Implications From MS

Therapeutic development in MS has focused on preventing immune cell trafficking into the CNS and depleting pathogenic B‐ and T‐cell populations [66, 67]. While trials of direct anti‐cytokine agents in MS have had more limited success compared to RA, they provide valuable data on the complexities of targeting these pathways in a CNS‐specific inflammatory context [68]. Basically, MS illustrates that compartmentalized CNS inflammation can preferentially present with fatigue, psychomotor slowing, and cognitive impairment, rather than purely affective symptoms [69]. These same symptom domains also show robust associations with inflammatory biomarkers in MDD [70]. Consequently, clinical trials should prioritize these specific dimensions (e.g., fatigue, cognitive performance, psychomotor speed) as key endpoints, rather than focusing solely on overall depression severity scores.

### 3.3 Systemic Lupus Erythematosus (SLE)

#### 3.3.1 SLE Pathogenesis and IL Profile

SLE is the archetypal systemic autoimmune disease, characterized by a profound loss of self‐tolerance, leading to the production of a vast array of autoantibodies against nuclear components. These autoantibodies form immune complexes that deposit in various tissues—including the kidneys, skin, and brain—triggering widespread inflammation and damage [71]. Type I interferons (especially IFN−α) are considered a central pathogenic driver [72, 73]. IL‐6 levels are often elevated and correlate with disease activity, and the IL‐17/IL‐23 axis contributes to organ damage [74]. A defining feature of SLE is the “IL‐10 paradox”: despite being a canonical anti‐inflammatory cytokine, IL‐10 levels are elevated in active SLE and correlate positively with disease activity [75]. This is because in the context of SLE, IL‐10's potent B‐cell stimulating effects—promoting their survival and autoantibody production—outweigh its other anti‐inflammatory functions, thus perpetuating the disease [75].

#### 3.3.2 Contrasts and Overlaps With MDD

SLE involves high‐grade, multi‐organ inflammation driven by a fundamental breakdown in B‐cell tolerance, a pathology far more severe and distinct from that of MDD [71]. The paradoxical, pathogenic role of IL‐10 in SLE is a critical contrast to its proposed insufficient but protective role in MDD. Nevertheless, important overlaps exist. IL‐6 and Type I IFNs are key mediators in both conditions [76]. The high prevalence of neuropsychiatric lupus (NPSLE), which includes severe depression, anxiety, and psychosis, provides another direct link between systemic immune dysregulation and severe psychiatric manifestations [77].

#### 3.3.3 Therapeutic Implications From SLE

The success of anifrolumab, an antibody blocking the Type I IFN receptor, validates targeting core cytokine pathways in SLE [78]. The IL‐10 paradox in SLE serves as a critical cautionary tale for the entire field of immunopsychiatry. It shatters any simplistic “pro‐ vs. anti‐inflammatory” dichotomy and demonstrates that the function of a cytokine is entirely context dependent. This warns against simplistic therapeutic strategies (e.g., “give IL‐10 to depressed patients”) and underscores the need for a deep, system‐level understanding of the specific immune context before intervening. Table 2 summarized the comparative immunophenotypes of MDD and RA.

**TABLE 2:** Comparative immunophenotypes of major depressive disorder and rheumatoid arthritis.

Feature	Major depressive disorder	Rheumatoid arthritis	Multiple sclerosis	Systemic lupus erythematosus	References
Key Pro‐inflammatory ILs	IL‐6, IL−1β, TNF−α, IL‐18	IL‐6, TNF−α, IL‐17, IL−1β	IL‐17, IFN−γ, IL−1β, IL‐6, IL‐12/23	Type I IFN, IL‐6, IL‐17, BAFF	[79]
Key Anti‐inflammatory ILs	IL‐10 (insufficient/dysregulated)	IL‐10, IL‐4	IL‐10 (protective)	IL‐10 (paradoxically pathogenic)	[80]
Dominant Immune Response	Innate dominant	Adaptive (Th1/Th17)	Adaptive (Th1/Th17)	Adaptive (B‐cell/Type I IFN)	[52, 53].
Magnitude of Systemic Inflammation	Low	High	Low‐to‐Moderate	High	[49]
Primary Locus of Inflammation	Systemic & CNS	Synovial Joints	Central Nervous System	Multi‐organ (kidney, skin, CNS)	[81, 82]
Evidence for CNS Inflammation	PET, CSF, Post‐mortem	Indirect (systemic cytokines)	Direct, high‐grade	Direct (NPSLE)	[15, 21, 83, 84]
Common Neuropsychiatric Symptoms	Anhedonia, fatigue, Cognitive dysfunction	High rates of depression, fatigue	High rates of depression and fatigue.	High rates of depression, anxiety, psychosis	[82]
Efficacy of Anti‐IL Therapies	Emerging/Mixed (requires stratification)	High (Anti‐TNF, Anti‐IL‐6R)	Limited for direct anti‐ILs	High (Anti‐IFNR, Anti‐BAFF)	[57]

### 3.4 Synthesis Across Autoimmune Comparators and Bidirectional Model of MDD‐Inflammation

**FIGURE 2:**
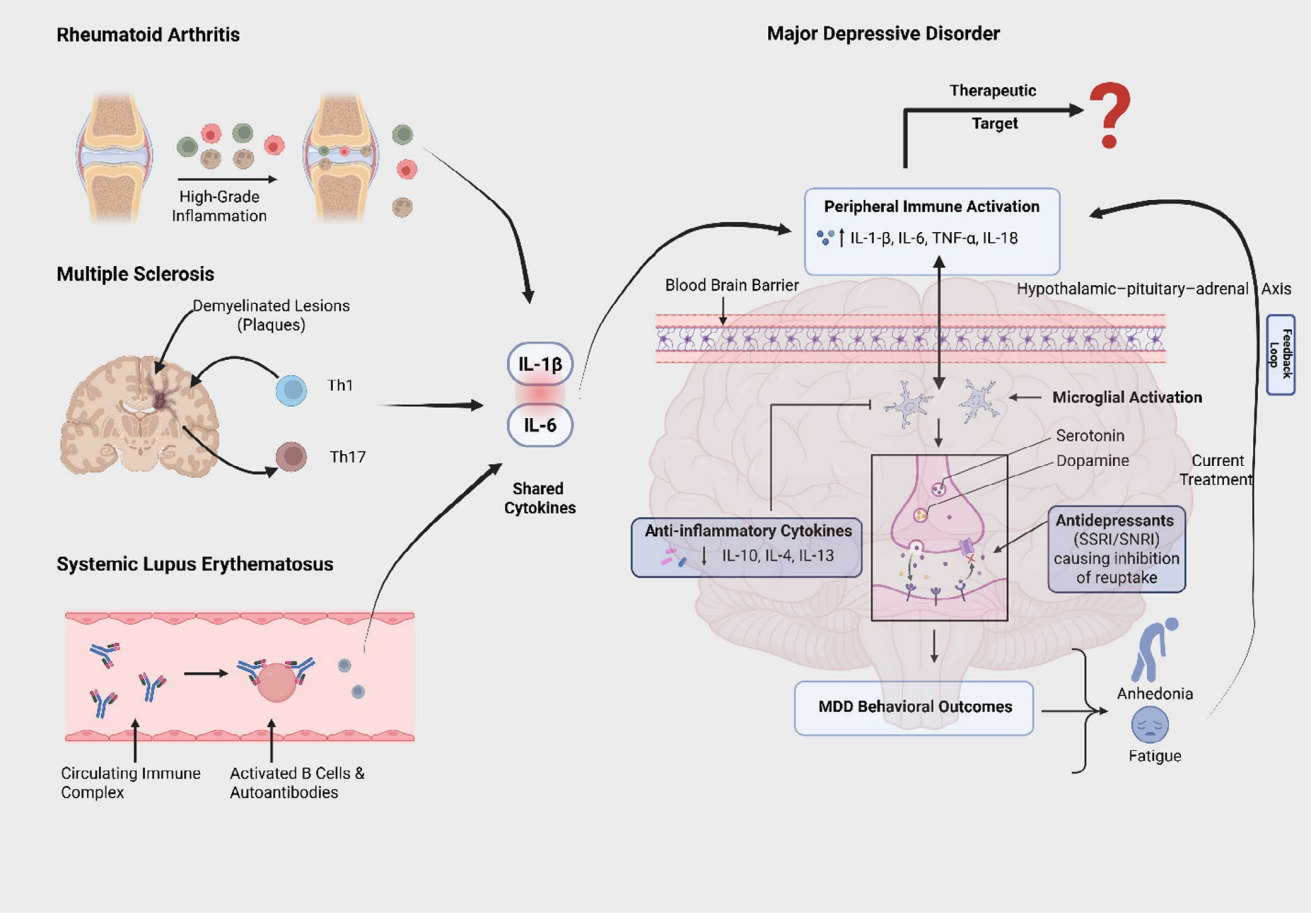
A conceptual comparative framework linking MDD with RA, MS, and SLE. The figure summarizes: (i) shared cytokines (IL‐1β and IL‐6); (ii) divergent immune contexts (e.g., high‐grade synovial inflammation in RA, CNS‐compartmentalized Th1/Th17 inflammation in MS, and B‐cell axis in SLE); (iii) therapeutic strategies derived for inflammatory MDD; and (iv) the bidirectional depression–inflammation loop. In this loop, elevated cytokines impact mood‐regulating circuits, while depressive symptom clusters—via stress system activation and behavioral changes—feedback to sustain systemic and CNS inflammation.

RA, MS, and SLE repeatedly implicate IL‐1β and IL‐6 yet differ in dominant drivers and tissue compartments. For MDD, this supports a model in which shared cytokines engage core neuroimmune mechanisms, while disease‐context features (e.g., Th17 skewing in MS or type I interferon/B‐cell dominance in SLE) help prioritize actionable targets in specific patient subgroups. It is also important to emphasize that these shared cytokine signatures operate within a bidirectional depression–inflammation loop, rather than a purely unidirectional “inflammation‐causes‐depression” model. Pro‐inflammatory mediators such as IL‐1, IL‐6, and TNF can alter monoamine turnover, glutamate transmission, and neuroplasticity, which can precipitate or worsen depressive symptoms [29, 85]. In parallel, depressive states marked by chronic stress exposure, HPA‐axis dysregulation, and adverse health behaviors can reactivate innate and adaptive immune pathways and sustain cytokine release [7, 86]. Figure 2 schematically integrates this reciprocal architecture by showing how a common cytokine milieu arising from RA, MS or SLE can both drive, and be reinforced by depressive symptomatology in susceptible individuals.

## 4 Diagnostic and Therapeutic Implications for MDD

### 4.1 Biomarker Potential

The identification of a reliable biomarker to pinpoint the “inflammatory MDD” subtype is a holy grail for the field. While promising, the clinical use of interleukins as diagnostic tools faces several hurdles. IL‐6 and CRP are the most consistently replicated peripheral biomarkers of inflammation in MDD [14, 87]. High‐sensitivity CRP (hs‐CRP), a stable and easily measured downstream indicator of IL‐6 activity, has already been used successfully as a stratification tool in clinical trials, with a cutoff like hs‐CRP > 3 mg/L often used to define an “inflamed” subgroup [88]. IL−1β and IL‐18, these inflammasome‐related cytokines are also strongly implicated and may help to define more specific mechanistic subtypes of depression [89]. The path forward likely involves moving beyond a single biomarker to utilizing multi‐marker panels or inflammatory ratios. For example, the IL‐6/IL‐10 ratio may provide a more nuanced picture of the overall balance between pro‐ and anti‐inflammatory forces than either cytokine alone, offering a more robust “inflammatory signature” [38, 39]. The primary challenge is the immense heterogeneity of MDD. As inflammation is only present in a subgroup, any potential biomarker signal will be diluted in unselected patient populations, leading to inconsistent results. Another challenge is distinguishing whether an elevated cytokine level is a stable trait marker of vulnerability or a transient state marker of a current depressive episode. Finally, while strong correlations are emerging, peripheral blood levels of cytokines may not always perfectly reflect the specific neuroinflammatory processes occurring within the CNS. Beyond circulating cytokines, incorporating cell‐based immune metrics (e.g., phenotyping of monocyte, T‐cell and NK‐cell subsets, activation markers, or ex vivo functional responses) may provide complementary information and help delineate inflammatory MDD subtypes with greater precision [90].

### 4.2 Therapeutic Targeting

The goal of this research is to develop novel treatments. The strategies being explored range from repurposing existing drugs to lifestyle interventions and developing novel molecules.

#### 4.2.1 Repurposing Existing Anti‐Cytokine Drugs

The most direct approach is to test the efficacy of biologic drugs already approved for autoimmune diseases. Trials with TNF inhibitors like Infliximab have yielded mixed results. A pivotal early study found that infliximab was effective, but *only* in the subgroup of depressed patients who had high baseline inflammation (high CRP) [91]. This finding was instrumental in establishing the critical principle of patient stratification. There are no interleukin‐targeting drugs currently approved for MDD, but several IL antagonists—especially those targeting IL‐1β and IL‐6—have shown promise in clinical and preclinical studies (Table 3). IL‐18, IL‐10, IL‐4, and IL‐13 are being explored, but evidence is still emerging.

**TABLE 3:** Anti‐inflammatory agents studied in major depressive disorder.

Drug	Target	Evidence in MDD	References
Anakinra	IL‐1 receptor antagonist	Reduced depressive symptoms in patients with high inflammation (CRP > 3 mg/L)	[91]
Tocilizumab	IL‐6 receptor blocker	Pilot studies show mood improvement in treatment‐resistant depression	[92]
Canakinumab	IL‐1β monoclonal antibody	Used in cardiovascular trials; secondary analysis suggests mood benefits	[93]
IL‐18BP (experimental)	IL‐18 binding protein	Preclinical models show reduced neuroinflammation and improved mood	[94]
IL‐10 gene therapy	IL‐10 agonist	Animal studies show antidepressant‐like effects via anti‐inflammatory pathways	[39]
Infliximab	TNF‐α monoclonal antibody	Improved motivation and reward processing in high‐CRP MDD patients	[27, 91]
NSAIDs	COX‐2	No Clinical Benefit yet	[95]

Notably, some anti‐inflammatory strategies including NSAIDs/COX‐2 inhibitors and minocycline show variable efficacy across trials; for example, a large treatment‐resistant depression add‐on minocycline trial did not outperform placebo [96], and meta‐analyses highlight heterogeneity across agents and study designs [97, 98]. Likely drivers include enrolling unselected MDD samples (only a subset is inflamed), inconsistent CNS target engagement, and reliance on global depression scales that may dilute domain‐specific benefits [99, 100].

### 4.3 Clinical Implications

The cumulative evidence linking interleukins to MDD heralds a paradigm shift in clinical psychiatry, moving beyond a purely monoamine‐centric model toward a more biologically nuanced understanding of the disorder. The most significant clinical implication is the validation of an “inflammatory subtype” of depression, suggesting that for a subset of patients, particularly those resistant to conventional antidepressants, the primary therapeutic target may be the immune system. This necessitates a move toward precision medicine, where biomarkers such as hs‐CRP could be integrated into routine clinical practice to stratify patients and guide treatment decisions [88, 91]. Most studies in Table 3 are proof‐of‐concept and differ in enrolment (inflamed vs. unselected), dosing, and endpoints. The infliximab TRD RCT (*n* = 60) was randomized, double‐blind, placebo‐controlled with three 5 mg/kg IV infusions over 6 weeks; overall efficacy was null, but benefit emerged in participants with hs‐CRP > 5 mg/L, establishing biomarker‐enriched designs [91]. The INSIGHT protocol applies the same logic to IL‐6 blockade: ~50 ICD‐10 depressed participants with low‐grade inflammation (hs‐CRP ≥ 3 mg/L) receive single‐dose IV tocilizumab versus saline, with behavioral/cognitive testing at Days 7/14/28 and a primary endpoint focused on somatic symptoms at ~Day 14 [92]. By contrast, small‐molecule anti‐inflammatories are often short adjunctive trials without immune stratification; celecoxib augmentation has shown mixed outcomes, including a negative 6‐week vortioxetine+celecoxib RCT [95]. Finally, large canakinumab cardiovascular RCTs demonstrate sustained IL‐1β pathway suppression with quarterly dosing in hs‐CRP‐enriched patients, but were not designed to test depression endpoints—highlighting the need for dedicated psychiatric trials that pre‐specify symptom‐domain outcomes (anhedonia, fatigue, cognition) and track inflammatory target engagement [101].

### 4.4 Challenges

Translating immunopsychiatry into routine clinical practice is promising but challenging. Success depends on overcoming several key hurdles. Patient stratification remains the most critical: future trials must move beyond a one‐size‐fits‐all approach and use biomarkers like hs‐CRP to identify depressed patients with inflammation who are most likely to respond. Target selection is another unresolved issue, as the optimal point in the inflammatory cascade—whether a single cytokine like IL‐6, an upstream hub like NLRP3, or a downstream pathway—requires further investigation. The blood–brain barrier presents a pharmacologic challenge, since large‐molecule biologics rarely penetrate the CNS. While peripheral effects may suffice, developing brain‐penetrant small molecules such as JAK or NLRP3 inhibitors is a major goal. Long‐term safety also demands attention, as chronic immunomodulation carries risks like increased susceptibility to infection. The risk–benefit balance of using these potent agents for MDD must be carefully evaluated.

### 4.5 Future Directions

Advancing immunopsychiatry will require breaking the bidirectional cycle in which inflammatory signaling drives depressive symptoms, depressive states in turn activate HPA‐axis and SNS pathways and promote maladaptive lifestyle factors, and this self‐reinforcing loop progressively undermines monoaminergic antidepressant efficacy and fosters chronicity. This may involve combining immunomodulators with conventional treatments to restore neurotransmitter function. Validating biomarker panels like IL‐6, CRP, and IL‐18 is essential for identifying responsive patients. Developing brain‐penetrant small molecules targeting upstream regulators such as NLRP3 remains a key therapeutic goal. Additionally, large‐scale trials are needed to standardize lifestyle interventions like diet and exercise as viable treatments for inflammatory depression.

## 5 Conclusion

This review has synthesized a substantial body of evidence establishing that dysregulation of the immune system, driven by key interleukins, is a critical pathophysiological component in a significant subgroup of individuals with MDD. This immune dysregulation is not an epiphenomenon; it directly impacts virtually every core neurobiological domain implicated in depression, including monoamine neurotransmitter metabolism, HPA axis function, adult neurogenesis, and synaptic plasticity. The comparative analysis of MDD alongside classical autoimmune diseases like RA, MS, and SLE has proven to be an exceptionally fruitful endeavor. This approach validates shared inflammatory pathways, such as those involving IL‐6, as legitimate therapeutic targets for the mood and vegetative symptoms that cut across these disorders. Most pragmatically, the therapeutic journey of immunology and neurology from broad immunosuppressants to highly targeted biologics and oral small molecules provides a clear and actionable roadmap for clinical translation in psychiatry, offering invaluable lessons in target selection, biomarker development, and clinical trial design.

## Author Contributions

**Adnan Akif:** data curation, formal analysis, visualization, investigation, writing – original draft. **Mohammad Fahim Kadir and Md. Rabiul Islam:** conceptualization, methodology, investigation, supervision, resources, writing – review and editing.

## Ethics Statement

It was an analysis of online available aggregate data. No Ethical approval was needed.

## Conflicts of Interest

The authors declare no conflicts of interest.

## Data Availability

Data sharing not applicable to this article as no datasets were generated or analyzed during the current study.
